# Balancing Immunity: GSK-3’s Divergent Roles in Dendritic Cell-Mediated T-Cell Priming and Memory Responses

**DOI:** 10.3390/ijms26136078

**Published:** 2025-06-25

**Authors:** Chunmei Fu, Tianle Ma, Li Zhou, Qing-Sheng Mi, Aimin Jiang

**Affiliations:** 1 Center for Cutaneous Biology and Immunology, Department of Dermatology, Henry Ford Health, Detroit, MI 48202, USA; cfu1@hfhs.org (C.F.); lzhou1@hfhs.org (L.Z.); qmi1@hfhs.org (Q.-S.M.); 2 Immunology Program, Henry Ford Cancer Institute, Henry Ford Health, Detroit, MI 48202, USA; 3 Department of Medicine, College of Human Medicine, Michigan State University, East Lansing, MI 48824, USA; 4 Department of Computer Science and Engineering, School of Engineering and Computer Science, Oakland University, Rochester, MI 48309, USA; tianlema@oakland.edu; 5 Department of Internal Medicine, Henry Ford Health, Detroit, MI 48202, USA

**Keywords:** glycogen synthase kinase-3, dendritic cells, β-catenin, vaccines, cross-priming, CD8 T-cell memory, cancer immunotherapy

## Abstract

Glycogen synthase kinase-3 (GSK-3)—particularly the GSK-3β isoform—plays a pivotal role in regulating dendritic cell (DC) functions, including maturation, cytokine production, and antigen presentation. In immature DCs, GSK-3β is continuously active, and its inhibition has been shown to enhance DC maturation and function. As a key upstream kinase of β-catenin, GSK-3 inhibition activates β-catenin in both human and murine DCs—a pathway traditionally linked to its immunomodulatory effects. However, our recent findings challenge this paradigm by uncovering β-catenin-independent, dual roles of GSK-3β in DCs. Our study reveals that while GSK-3β enhances DC-mediated cross-priming of CD8 T cells, it concurrently impairs the generation of memory CD8 T cells. These findings have significant implications for vaccine development and cancer immunotherapy, where both effective T-cell priming and durable memory responses are critical. This mini-review provides an in-depth analysis of mechanistic insights into GSK-3β’s paradoxical functions and discusses potential strategies to fine-tune GSK-3 activity for optimized immunotherapeutic outcomes.

## 1. Introduction

Dendritic cells (DCs) are professional antigen-presenting cells (APCs) that play a central role in linking innate and adaptive immunity. They process and present antigens to T cells, typically presenting exogenous antigens via MHC class II to CD4 T cells, and endogenous or cytosolic antigens via MHC class I to CD8 T cells. Notably, DCs are also uniquely capable of cross-presentation. However, DCs are also uniquely capable of cross-presentation—a process in which exogenous antigens are routed into the MHC class I pathway to activate naïve CD8 T cells, a mechanism known as cross-priming [1,2,3]. This function is particularly relevant for initiating immune responses against viruses and tumors that do not directly infect DCs. Among APCs, DCs are especially effective at cross-presenting tumor antigens and priming tumor-specific CD8 T cells to mediate tumor control [4,5,6,7,8,9]. This specialized function makes DCs attractive targets for immunotherapeutic approaches, including DC-based vaccines designed to enhance anti-tumor immunity. However, despite promising preclinical results, clinical trials using DC-based vaccines have achieved only limited success [10,11,12,13,14,15]. One major challenge is that these vaccines often depend on host DCs to present antigens and initiate robust T-cell responses [16,17,18]; tumors frequently impair host DC function to evade immune surveillance [19,20,21,22,23]. Consequently, tumor-induced DC dysfunction represents a major barrier to the effective development of DC-based cancer vaccines.

Tumor-induced activation of β-catenin in DCs has been shown to impair their function in mediating anti-tumor CD8 T-cell responses [24,25,26,27], further supporting β-catenin as a critical negative regulator of DC function [28,29]. However, β-catenin in DCs also plays a positive role in the maintenance of primed CD8 T cells and memory responses [30], indicating its context-dependent roles, limiting its utility as a therapeutic target for enhancing vaccine efficacy.

Glycogen synthase kinase-3 (GSK3), a conserved serine/threonine kinase best known for its involvement in the Wnt/β-catenin signaling pathway, has been extensively studied in various biological functions, including metabolism, neurobiology, and immune regulation [31,32]. Recent studies have highlighted its importance in T-cell activation, leading to the development of small-molecule inhibitors of GSK-3 designed to enhance CD8 T-cell responses for cancer immunotherapy [33]. Similarly, GSK-3—particularly the GSK-3β isoform that predominates in immune cells—regulates various signaling pathways that govern DC maturation, activation, and antigen presentation [34,35,36]. Pharmacological inhibition of GSK-3β stabilizes β-catenin and has been shown to enhance DC maturation, pro-inflammatory cytokine production, and DC function [34,36,37,38,39,40].

Nevertheless, the specific roles of GSK-3β in cross-priming versus memory formation have remained unclear. Our group addresses this gap using genetic models to dissect the function of GSK-3β in DCs (Fu et al. 2024 [41]). Surprisingly, we find that GSK-3β enhances cross-priming independently of β-catenin, while concurrently limiting CD8 memory T-cell responses. These findings challenge the prevailing view that GSK-3 acts through β-catenin and suggest distinct roles for GSK-3β in different phases of the CD8 T-cell response.

In this mini-review, we discuss our new findings from Fu et al. 2024 [41] in the context of existing literature and their implications for therapeutic strategies aiming to modulate DC function in cancer immunotherapy.

## 2. GSK-3 in DCs, a β-Catenin-Related Affair

Glycogen synthase kinase-3 (GSK-3), particularly the GSK-3β isoform, plays multifaceted and context-dependent roles in DC biology [42]. Among its most well-characterized functions is its role in the canonical Wnt/β-catenin signaling pathway. In the absence of Wnt ligands, GSK-3 forms a destruction complex with axin, adenomatous polyposis coli, and β-catenin, where it actively phosphorylates β-catenin, targeting β-catenin for ubiquitination and subsequent proteasomal degradation [43,44]. When Wnt ligands bind to Frizzled receptors, GSK-3 activity is inhibited, allowing β-catenin to accumulate and translocate to the nucleus and interact with TCF/LEF transcription factors to initiate transcriptional programs involved in cell survival, proliferation, and immune regulation. Beyond Wnt signaling, GSK-3 is involved in other key signaling pathways, including PI3K/AKT and NF-κB, further highlighting its central role in immune regulation.

**Figure 1:**
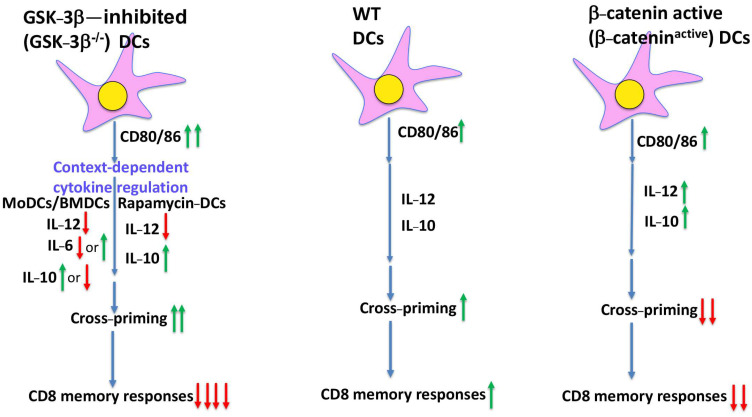
Schematic overview of the context-dependent regulation of DC functions by GSK-3β, in comparison to β-catenin. Inhibition of GSK-3β promotes DC maturation, characterized by upregulation of co-stimulatory molecules such as CD80 and CD86. GSK-3β inhibition also modulates cytokine production—including IL-12, IL-6, and IL-10—in a context-dependent manner. In DC-targeted vaccination settings, conditional deletion of GSK-3β in DCs (CD11c-GSK-3β^−/−^ mice) enhances the cross-priming of antigen-specific CD8 T cells during the primary phase but fails to generate robust memory CD8 T-cell responses. These findings indicate that GSK-3β in DCs plays divergent roles in regulating primary versus memory CD8 T-cell responses. The distinct phenotype of CD11c-β-catenin^active^ mice compared to CD11c-GSK-3β^−/−^ mice supports the idea that GSK-3β regulates DC function through β-catenin-independent mechanisms.

GSK-3 is constitutively active in immature DCs and inhibits the spontaneous maturation of immature human monocyte-derived DCs (MoDCs) [34]. Pharmacological inhibition of GSK-3 using various small-molecule inhibitors such as lithium chloride, CHIR99021, or SB216763 leads to activation of β-catenin and modulates the induction of inflammatory cytokines, including IL-12, IL-6, TNF-α, and IL-10 [34]. In murine bone marrow–derived DCs (BMDCs) and splenic DCs, similar patterns have been observed (Figure 1). Alessandrini et al. reported that GSK-3β was constitutively active in immature DCs but became inhibited following exposure to maturation stimuli such as lipopolysaccharide (LPS), coinciding with β-catenin accumulation and activation [36]. Notably, the inhibition of GSK-3β in this setting enhanced the expression of costimulatory molecules (CD80, CD86, CD40), improved antigen presentation, and increased IL-2 secretion by T cells, suggesting a role in enhancing DC immunogenicity [36]. Furthermore, GSK-3β appears to exert a pro-apoptotic effect in DCs, as its inhibition improves DC survival [45], suggesting that targeted modulation of GSK-3β may enhance DC function and T-cell activation.

However, GSK-3β’s effects on DC maturation and function are not uniform and vary depending on the differentiation context and external stimuli (Figure 1). For instance, its inhibition has also been shown to suppress DC maturation in response to specific stimuli such as anthrax edema toxin [46]. In certain settings, such as DCs treated with LPS in the presence of the mTOR inhibitor rapamycin, GSK-3 remains active and β-catenin levels are markedly reduced [38]. In this context, Turnquist et al. showed that GSK-3 inhibition suppressed IL-12 production, indicating that GSK-3 activity may be necessary for optimal cytokine output under certain immunomodulatory conditions [38]. Similarly, in human MoDCs exposed to the fungal pathogen *Aspergillus fumigatus*, GSK-3 activity was shown to modulate inflammatory cytokine responses, further supporting its context-specific regulatory role [47].

GSK-3β also regulates the production of the anti-inflammatory cytokine IL-10 in DCs (Figure 1). For example, Wang et al. demonstrated that active GSK-3β suppressed IFN-β-mediated IL-10 induction, whereas GSK-3β inhibition augmented IL-10 production [39], consistent with β-catenin’s known role in promoting IL-10 induction [30]. In another study, Fas signaling-induced IL-10 production in DCs was attributed to GSK-3 inhibition and subsequent β-catenin activation [48]. Furthermore, inhibiting GSK-3 during the differentiation of BMDCs impaired their ability to polarize Th2 cells, likely due to altered costimulatory molecule expression (CD80, CD86) and cytokine induction [37].

Beyond cytokine regulation, GSK-3β regulates DC immunoregulatory function through modulation of indoleamine 2,3-dioxygenase (IDO), an immunosuppressive enzyme critical for immune tolerance and T-cell suppression [40]. Pharmacological blockade of GSK-3β downregulated IDO expression, enhanced CD8 T-cell proliferation and cytotoxicity, and improved the efficacy of DC vaccines in preclinical models [40]. These findings suggest that GSK-3β blockade could be leveraged to enhance anti-tumor immunity. However, a recent study using human DCs challenged this paradigm, reporting that constitutively active GSK-3β promoted DC development and maturation, and enhanced the immunogenicity of DC vaccines [49]. These seemingly contradictory observations underscore the highly context-dependent effects of GSK-3β on DC biology.

It should be noted that most of the current understanding of GSK-3 (particularly GSK-3β) in DCs derives from studies employing pharmacological inhibitors. While these approaches have provided valuable insights, they are limited by off-target effects and often fail to distinguish between the contributions of GSK-3α and GSK-3β isoforms. Moreover, although β-catenin activation frequently accompanies GSK-3(β) inhibition, whether β-catenin mediates the function of GSK-3(β) has not been directly tested, highlighting the need for more precise genetic approaches. To address this critical gap, we employed conditional knockout models to selectively delete GSK-3β in DCs, enabling a more precise dissection of its roles in DC development, function, and immune regulation [41].

## 3. Unexpected Dual Roles of GSK-3 in DC Vaccine-Induced CD8 T-Cell Responses Independent of β-Catenin

To address conflicting reports regarding the role of GSK-3 in DCs, we (Fu et al. 2024) employed a genetic approach to dissect GSK-3β function specifically in DCs [41]. We bred floxed GSK-3β mice with CD11c-Cre mice to generate CD11c-GSK-3β^−/−^ mice with DC-specific deletion of GSK-3β. As inhibition of GSK-3β is thought to activate β-catenin, it is expected that deletion of GSK-3β in DCs would photocopy β-catenin activation and impair DC vaccine-induced CD8 T-cell responses. Surprisingly, this was not the case.

Contrary to expectations, we observed that CD11c-GSK-3β^−/−^ mice exhibited enhanced instead of impaired cross-priming upon immunization with anti-DEC-205OVA—a phenotype that is opposite to that observed with CD11c-β-catenin^active^ mice (Fu et al. 2024 [41]). Even more unexpectedly, memory CD8 T-cell responses were almost abrogated in CD11c-GSK-3β^−/−^ mice upon anti-DEC-205OVA vaccination despite augmented cross-priming. These findings reveal a striking dissociation: While GSK-3β-deficient DCs are capable of initiating strong effector CD8 T-cell responses, they are profoundly impaired in their ability to generate/support long-lived memory T-cell formation (Figure 1).

Interestingly, we had previously shown that CD11c-β-catenin^−/−^ mice similarly exhibit enhanced cross-priming but impaired memory CD8 T-cell responses [30], suggesting that GSK-3β may function independently of β-catenin (Figure 1). Supporting this idea, we further demonstrated that specific deletion of GSK-3β in DCs did not lead to accumulation/activation of β-catenin protein, as assessed by Western blot and flow cytometry [41]. As previous studies in T cells have shown that GSK-3β regulates the expression of the immune checkpoint molecules, including PD-1 and Lag3, we also examined these molecules and showed that the expression of immune checkpoint molecules PD-L1, PD-L2, Lag3, and Tim-3 was not significantly altered in GSK-3β^−/−^ DCs [41]. This pattern is quite different from that of β-catenin^active^ DCs, which have been shown to exhibit elevated PD-L2 and Tim-3 but diminished PD-L1 [50]. These differences further argue that GSK-3β functions in DCs through mechanisms distinct from β-catenin signaling.

To further dissect the divergence between GSK-3β- and β-catenin-regulated pathways, we further carried out single-cell RNA sequencing (scRNA-seq) experiments to analyze the transcriptional profiles of GSK-3β^−/−^ versus β-catenin^active^ DCs [41]. These scRNA-seq data identified 13 distinct cell populations, including monocyte-derived DCs (MoDCs), conventional DCs (cDCs), plasmacytoid DCs (pDCs), IFN-producing killer DCs, transitional DCs, migratory DCs, and B cell-like pDCs. Not surprisingly, GSK-3β^−/−^ and β-catenin^active^ DCs exhibit intriguing differences in their distribution of these DC populations, suggesting that GSK-3β inhibition and β-catenin likely regulate DC differentiation independently. For example, while GSK-3β inhibition likely plays a negative role as deletion of GSK-3β leads to reduced number of cDC1s, β-catenin^active^ DCs exhibited increased cDC1s, suggesting a potential positive role for β-catenin in cDC1 development. In contrast, deletion of GSK-3β but not β-catenin activation leads to substantially increased pDCs, indicating that GSK-3β but not β-catenin plays a critical role in pDC development. For IFN-producing killer DCs, while GSK-3β^−/−^ DCs exhibited a much reduced number of these DCs, β-catenin^active^ DCs do not affect this population, suggesting that only GSK-3β plays a role in regulating their development. Overall, these findings suggested that GSK-3β and β-catenin likely regulate DC development and differentiation independently.

We also directly compared gene expression patterns between GSK-3β^−/−^ DCs and β-catenin^active^ DCs using scRNA-seq data, uncovering substantial differences in their gene expression patterns. Strikingly, this analysis identified only minimal overlap between GSK-3β^−/−^ DCs and β-catenin^active^ DCs in differentially expressed genes (DEGs)—3.9% among upregulated and 0.8% among downregulated genes—indicating distinct transcriptional programs. Pathway enrichment analysis also revealed different top upregulated/downregulated pathways, consistent with the contrasting checkpoint molecule profiles observed between the two genotypes. Pathway analysis additionally reveals that GSK-3β^−/−^ and β-catenin^active^ DCs exhibit completely different top upregulated/downregulated pathways. Furthermore, different expression patterns of immune checkpoint molecules (PD-L1/L2, Tim-3, and Lag3) are observed between GSK-3β^−/−^ and β-catenin^active^ DCs, consistent with their protein expression patterns [41,50]. Taken together, these scRNA-seq data indicate that GSK-3β deletion does not result in upregulation of β-catenin at the transcriptional or protein level.

This uncoupling of GSK-3β and β-catenin challenges the prevailing assumption that GSK-3’s immune effects are mediated through β-catenin and instead suggests that GSK-3β governs distinct phases of the CD8 T-cell response through independent mechanisms. Previous studies have shown that deletion of β-catenin in DCs achieves opposite functions in cross-priming versus memory CD8 T-cell responses through IL-10 [30]. However, GSK-3 blockade in DCs is shown to negatively regulate IL-10 [37,39,49,51,52,53], opposite to reduced IL-10 induction in β-catenin^−/−^ DCs [30], suggesting that IL-10 is unlikely to mediate GSK-3β’s dual functions in regulating cross-priming and memory CD8 T-cell responses.

To elucidate the underlying mechanisms of how GSK-3β in DCs exerts opposite functions in regulating vaccine-induced CD8 T-cell responses, we performed scRNA-seq experiments to compare gene expression of primed antigen-specific CD8 T (OTI) cells at days 4 and 10 post-anti-DEC-205OVA immunization. At day 4 post-immunization, primed OTI cells exhibited a higher effector index score in CD11c-GSK-3β^−/−^ mice compared to primed OTI cells in WT mice, consistent with enhanced cross-priming. However, by day 10 post-immunization, these primed OTI cells exhibited lower effector index scores and lower memory index scores than primed OTI cells in their WT counterparts. Furthermore, memory cell populations, which are identified by high expression of memory gene markers, are greatly reduced in CD11c-GSK-3β^−/−^ mice at day 10 post-immunization, suggesting that GSK-3β^−/−^ DCs might be impaired in their function in generating/maintaining memory CD8 T cells.

Further pathway analyses revealed that genes involved in effector and memory function-related IL-2/STAT5 signaling, TNF-α signaling, and IFN-γ responses are among the most significantly downregulated pathways in primed OTI cells in CD11c-GSK-3β^−/−^ compared to WT controls following anti-DEC-205OVA immunization. Mechanistically, GSK-3β has been shown to directly activate STAT5 [54], enhance NF-κB-mediated transcription downstream of TNF-α [55], and facilitate IFN-γ-induced STAT1 signaling [56]. These findings suggest that GSK-3β^−/−^ DCs may modulate OTI effector and memory responses through these signaling pathways independent of β-catenin. However, as GSK-3 functions within DCs, these effects are indirect and highlight the need for further investigation into the molecular mechanisms by which DC-intrinsic GSK-3β regulates T-cell programming.

Collectively, these findings support a model in which deletion of GSK-3β in DCs regulates transcriptional programs in primed antigen-specific CD8 T cells to augment their effector differentiation and function during the priming phase (at day 4) but impair the generation and/or maintenance of memory CD8 T cells, resulting in diminished memory responses. These dual functions are independent of β-catenin and likely involve distinct transcriptional and signaling programs that regulate the T-cell response over time. Further studies to elucidate the precise molecular mechanisms underlying GSK-3β-mediated modulation of DC function may provide new strategies to optimize DC-based vaccines by temporally manipulating GSK-3β activity to enhance both acute anti-tumor immunity and long-term immune protection.

## 4. Implications for Therapeutic Targeting of GSK-3 in Cancer Immunotherapies

**Figure 2:**
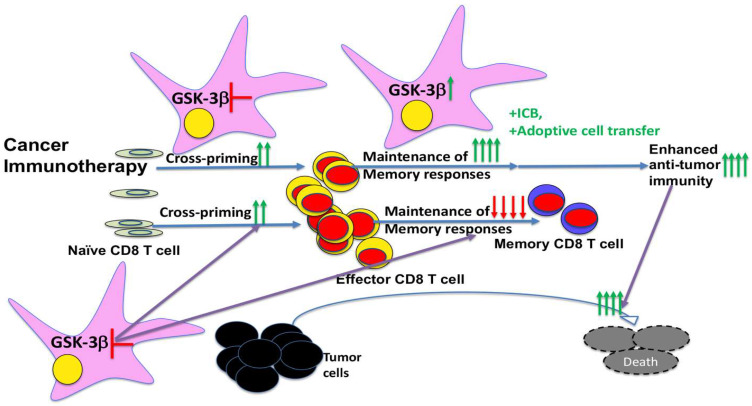
Schematic summary of the divergent roles of GSK-3β in DC-mediated CD8 T-cell responses and its therapeutic implications. Conditional deletion of GSK-3β in DCs (CD11c-GSK-3β^−/−^ mice) enhances the cross-priming of antigen-specific CD8 T cells during the primary responses but markedly impairs the generation of memory CD8 T cells. These opposing effects underscore the importance of temporally modulating GSK-3β activity in DCs to optimize immune outcomes. A strategic approach—transient inhibition of GSK-3β during the priming phase to amplify effector T-cell responses, followed by restoration of its activity during the memory phase—may offer a more effective path to fully harness the therapeutic potential of GSK-3β modulation to generate robust, durable anti-tumor immunity. This temporal modulation could also synergize with ICB or other immunotherapy strategies to improve anti-tumor efficacy.

Our new findings in Fu et al. (2024) [41], highlight GSK-3β as a pivotal regulator of DC-mediated immunity, with a dual role in promoting cross-priming while simultaneously limiting memory CD8 T-cell responses (Figure 2). This paradox presents both opportunities and challenges in the therapeutic modulation of GSK-3β in cancer immunotherapy.

Notably, we demonstrate that deletion of GSK-3β in DCs enhances cross-priming of antigen-specific CD8 T cells, resulting in more robust early effector responses. This suggests that transient inhibition of GSK-3β during the priming phase of DC vaccination could serve as a potent strategy to boost initial anti-tumor immunity. This strategy may be particularly advantageous in enhancing the efficacy of DC-based cancer vaccines, similar to a previous study showing improved DC vaccine efficacy by blocking β-catenin during the priming phase [30]. Given that small-molecule inhibitors of GSK-3 have been tested for cancer immunotherapy based on the role of GSK-3 in T cells [33]—where GSK-3 blockade leads to downregulation of inhibitory checkpoint molecules such as PD-1 and Lag3 [57,58,59]—GSK-3 blockade thus has the advantage of targeting both arms of the immune response (DCs and T cells) to synergistically improve anti-tumor efficacy.

However, we have demonstrated that GSK-3β deletion in DCs does not alter gene expression of PD-L1, PD-L2, Lag3, or Tim-3 at either the transcript or protein levels [41], in contrast to its effects in T cells. This indicates that GSK-3β likely functions in a context- and cell type-dependent manner. Notably, the enhancement of DC vaccine efficacy via GSK-3β inhibition appears to operate through pathways distinct from those targeted by conventional immune checkpoint blockade. Therefore, combining GSK-3β inhibition in DCs with ICB therapies (e.g., anti-PD-1 or anti-Lag3) could offer additive or synergistic effects to maximize therapeutic benefit.

Importantly, we (Fu et al. 2024) also reveal that while GSK-3β deficiency in DCs enhances early/primary CD8 T-cell responses, it impairs the ability to support the generation and/or maintenance of memory CD8 T cells (Figure 2), which are crucial for long-term immune protection against tumor recurrence. This finding mirrors earlier work showing that deletion of β-catenin in DCs similarly impairs memory CD8 T-cell responses despite augmented cross-priming [30]. The fact that both deletion of GSK-3β and β-catenin exert opposite functions in cross-priming and memory CD8 T-cell responses through distinct mechanisms suggests that the balance of primary effector responses versus memory CD8 T-cell generation is carefully calibrated to achieve optimal CD8 T-cell responses.

While GSK-3β inhibition during the priming phase may enhance dendritic cell activation, cross-presentation, and primary CD8 T-cell effector responses to improve early tumor control, sustained inhibition may be detrimental to the development and maintenance of memory T cells. Consistent with our findings on diminished memory responses, GSK-3β has been shown to promote critical signaling pathways such as NF-κB and IL-2/STAT5, both essential for the survival, homeostasis, and function of memory T cells [54,55]. Thus, strategies to preserve or re-activate GSK-3β during the memory phase may be necessary to sustain long-term protective immunity.

In the context of cancer immunotherapy, this has important implications. As DC-based vaccines and T-cell-directed therapies aim to generate both potent effector responses and durable memory responses against tumor recurrence, however, clinical outcomes have often been limited by poor memory T-cell persistence or functional exhaustion. The dual roles of GSK-3β suggest that a time-sensitive approach to its modulation may improve therapeutic efficacy: blocking GSK-3β transiently during the priming phase to boost primary CD8 T-cell responses leading to robust effector function, followed by reactivation or maintenance of GSK-3β activity during the memory phases to generate long-lived antigen-specific memory T cells (Figure 2). Such a temporal approach may be especially effective when combined with ICB or other immunotherapies designed to further improve durable anti-tumor immunity.

Future studies are warranted to understand how GSK-3β signaling in DCs influences the quality of CD8 T-cell memory across different vaccine platforms and tumor settings. Understanding this balance will be critical for designing rational combination therapies that maximize both primary and memory T-cell responses to control tumors.

In conclusion, understanding the temporal and cell-type-specific roles of GSK-3β will be critical for translating these findings into effective and durable cancer immunotherapies. Tailoring GSK-3β modulation to the immune phase and cellular context may ultimately unlock its full therapeutic potential in driving effective and durable anti-tumor responses.

## 5. Conclusions and Perspectives

Our recent findings by Fu et al. 2024 uncover a previously unrecognized dual role for GSK-3β in DC-mediated immunity, offering a nuanced view of how GSK-3β in DCs regulates CD8 T-cell responses [41]. Contrary to the current paradigm that GSK-3β exerts its effects through the canonical Wnt/β-catenin signaling pathway, we provide compelling evidence that GSK-3β likely operates through β-catenin-independent mechanisms in DCs. Specifically, our work reveals that deletion of GSK-3β in DCs promotes early CD8 T-cell priming while simultaneously constraining memory CD8 T-cell responses (Figure 1).

Importantly, we demonstrate in Fu et al. 2024 that deletion of GSK-3β in DCs enhances cross-priming and augments early effector CD8 T-cell responses without stabilizing β-catenin or altering immune checkpoint expression [41]. The minimal overlap in gene expression between DCs with active β-catenin versus GSK-3β-deficient DCs [41] further supports the conclusion that GSK-3β indeed exerts its function independently of β-catenin in DCs, reinforcing the context-dependent function of GSK-3β (Figure 1). Notably, the augmented cross-priming observed with GSK-3β-deficient DCs comes at the cost of diminished memory CD8 T-cell responses, indicating a phase-specific function of GSK-3β in regulating the immune response. These insights provide a mechanistic foundation for selectively modulating GSK-3β activity at different stages of the immune response to improve anti-tumor efficacy (Figure 2).

Together, these new findings highlight the potential of targeting GSK-3β to improve DC-based cancer vaccines. Temporally controlled inhibition of GSK-3β during the priming phase—while preserving or restoring its activity during the memory phase—could offer both potent immediate effector responses and durable, long-term immune protection (Figure 2). Our study thus advances our understanding of the complex, context-dependent functions of GSK-3β in immune regulation and sets the stage for future translational efforts to fine-tune GSK-3β activity in a context- and time-dependent manner for optimal immunotherapeutic benefit. Future studies should further define the molecular pathways downstream of GSK-3β that govern these distinct immune phases and explore pharmacological or genetic tools for precise temporal control of their activity in vivo.

## Data Availability

Not applicable.

## Abbreviations

The following abbreviations are used in this manuscript:

DCs	dendritic cells
APCs	antigen-presenting cells
ICB	immune checkpoint blockade
MoDCs	monocyte-derived DCs
BMDCs	bone marrow-derived DCs
GSK-3	glycogen synthase kinase-3
IDO	indoleamine 2,3-dioxygenase
LPS	lipopolysaccharide
